# Infection with the multidrug-resistant Klebsiella pneumoniae New Delhi metallo-B-lactamase strain in patients with COVID-19: *Nec Hercules contra plures?*

**DOI:** 10.3389/fcimb.2024.1297312

**Published:** 2024-04-16

**Authors:** Jarosław Janc, Natalia Słabisz, Anna Woźniak, Lidia Łysenko, Mariusz Chabowski, Patrycja Leśnik

**Affiliations:** ^1^ Department of Anaesthesiology and Intensive Therapy, Hospital of Ministry of the Interior and Administration, Wrocław, Poland; ^2^ Department of Microbiology, 4th Military Clinical Hospital, Wrocław, Poland; ^3^ Department of Nursing and Midwifery, Wroclaw Medical University, Wrocław, Poland; ^4^ Departament of Anaesthesiology and Intensive Care Unit, Wroclaw Medical University, Wrocław, Poland; ^5^ Department of Surgery, 4th Military Clinical Hospital, Wrocław, Poland; ^6^ Department of Clinical Surgical Sciences, Faculty of Medicine, Wrocław University of Science and Technology, Wrocław, Poland; ^7^ Department of Microbiology, Wroclaw Medical University, Wrocław, Poland

**Keywords:** coronavirus disease 2019, Klebsiella pneumoniae, metallo-B-lactamase, hospital-acquired infection, mortality

## Abstract

**Background:**

During the coronavirus disease 2019 (COVID-19) pandemic, in patients treated for SARS-CoV-2 infection, infections with the *Klebsiella pneumoniae* bacteria producing New Delhi metallo-B-lactamase (NDM) carbapenemase in the USA, Brazil, Mexico, and Italy were observed, especially in intensive care units (ICUs). This study aimed to assess the impact of *Klebsiella pneumoniae* NDM infection and other bacterial infections on mortality in patients treated in ICUs due to COVID-19.

**Methods:**

The 160 patients who qualified for the study were hospitalized in ICUs due to COVID-19. Three groups were distinguished: patients with COVID-19 infection only (N = 72), patients with COVID-19 infection and infection caused by *Klebsiella pneumoniae* NDM (N = 30), and patients with COVID-19 infection and infection of bacterial etiology other than *Klebsiella pneumoniae* NDM (N = 58). Mortality in the groups and chosen demographic data; biochemical parameters analyzed on days 1, 3, 5, and 7; comorbidities; and ICU scores were analyzed.

**Results:**

Bacterial infection, including with *Klebsiella pneumoniae* NDM type, did not elevate mortality rates. In the group of patients who survived the acute phase of COVID-19 the prolonged survival time was demonstrated: the median overall survival time was 13 days in the NDM bacterial infection group, 14 days in the other bacterial infection group, and 7 days in the COVID-19 only group. Comparing the COVID-19 with NDM infection and COVID-19 only groups, the adjusted model estimated a statistically significant hazard ratio of 0.28 (p = 0.002). Multivariate analysis revealed that age, APACHE II score, and CRP were predictors of mortality in all the patient groups.

**Conclusion:**

In patients treated for SARS-CoV-2 infection acquiring a bacterial infection due to prolonged hospitalization associated with the treatment of COVID-19 did not elevate mortality rates. The data suggests that in severe COVID-19 patients who survived beyond the first week of hospitalization, bacterial infections, particularly Klebsiella pneumoniae NDM, do not significantly impact mortality. Multivariate analysis revealed that age, APACHE II score, and CRP were predictors of mortality in all the patient groups.

## 1 Introduction

After the outbreak of COVID-19 in December 2019 in China, it rapidly spread throughout China and all around the world. Data presented by the World Health Organization show that more than 126 million cases of COVID-19 have been reported worldwide, of which one-fourth have been in the USA with the remainder in other countries, including European nations (Sun et al., 2020; Phannajit et al., 2021).

The SARS-CoV-2 virus infects the body and causes inflammatory changes in the vascular endothelium, activating the immune system and coagulation and causing organ damage, mainly to the lungs, brain, heart, and kidneys (Hekmatnia et al., 2022). Respiratory viruses, such as coronaviruses and influenza viruses, can cause acute damage to lung epithelial cells, allowing other pathogens to infiltrate the affected area (de Wit et al., 2016; Zhong et al., 2021).

This allows the subsequent invasion of other microorganisms, including bacteria and fungi. A hospital environment poses a risk of supra-infection with nosocomial strains. This is particularly true for patients treated in intensive surveillance and intensive care units (ICUs), where they may be exposed to multiple invasive therapeutic procedures. The latter require an integrated, multifaceted approach to preventing multidrug resistance (Pelfrene et al., 2021).

Due to elevated inflammatory parameters, which make it difficult to distinguish COVID-19 from secondary bacterial infections, the empirical use of broad-spectrum antibiotic therapy is recommended (Beović et al., 2020; Langford et al., 2020; Monnet and Harbarth, 2020; Rawson et al., 2020; Wan et al., 2020). This approach has led to the propagation of multidrug-resistant strains. During the COVID-19 pandemic, due to the large numbers of patients with COVID-19 in hospitals, the creation of temporary wards dedicated to the treatment of these patients and the hiring of additional staff often not trained to care for critically ill patients resulted in the spread of multidrug-resistant strains in hospitals.

*Klebsiella pneumoniae* belongs to the family *Enterobacteriaceae*, which is part of the normal human intestinal flora but can also be responsible for community- and healthcare-associated infections. Due to acquiring resistance genes, a rapid increase in resistance to penicillins and cephalosporins has been observed. Much more dangerous resistance to carbapenems has been reported with increasing frequency and geographical spread since the 1990s (Gupta et al., 2011; Munoz-Price et al., 2013).

Carbapenem-resistant *Enterobacterales* can be resistant to carbapenems as a result of various mechanisms. One of the representatives of this group, *Klebsiella pneumoniae*, produces metallo-beta-lactamases—enzymes that hydrolyze most beta-lactams, including carbapenems (Nordmann et al., 2011).

During the COVID-19 pandemic, in patients treated for SARS-CoV-2 infection, infections with the *Klebsiella pneumoniae* bacteria producing New Delhi metallo-B-lactamase (NDM) carbapenemase in the USA, Brazil, Mexico, and Italy were observed, especially in ICUs (Nori et al., 2020; Porretta et al., 2020; Fernández-García et al., 2022; Monteiro et al., 2023).

The *Klebsiella pneumoniae* NDM strain was first described in 2009, isolated from the urine of a patient in Sweden who had been hospitalized in India (Yong et al., 2009). In Poland, the first NDM infection was reported in 2015 (Sacha et al., 2012). The occurrence of NDM strains is associated with the likelihood of the development of resistance to all available antibiotics and the risk of transmitting resistance genes to various species of microorganisms (Queenan and Bush, 2007). The most significant problem is the production of carbapenemases due to the possibility of horizontal gene transfer between bacteria of the same or different species via plasmids. Currently, there are several types of carbapenemases, including KPC and metallo-B-lactamase enzymes, referred to as NDM (New Delhi metallo-B-lactamase, IMP, VIM) and OXY-48-like carbapenemases. Admission to an ICU and age over 70 years were found to be likely risk factors for infection with CR and ESBL *K. pneumoniae*, with increasing age being correlated with greater risk factors (Wang et al., 2023). Inappropriate antibiotic therapy treatment and extended ICU stays (15 days) were the most significant clinical and epidemiological factors (El Mekes et al., 2020).

Respiratory failure patients tend to have more severe illnesses than those with chronic obstructive pulmonary disease. The ICU is a common place to manage these patients because they frequently require mechanical ventilation and are more likely to become infected or colonized with CR *K. pneumoniae* in the airway.

Between September 2020 and September 2022, a rapid spread of *K. pneumoniae* NDM-type was observed in the wards of the 4th Military Clinical Hospital in Wroclaw, Poland. It concerned patients treated both in the ICU and wards dedicated to patients with COVID-19.

Staff caring for patients with COVID-19 were protected by required clothing, but contamination of this clothing by microorganisms and errors in hygiene procedures led to the spread of hospital-acquired infections in the hospital environment (Pratt et al., 2001).

The present study aimed to assess the impact of *K. pneumoniae* NDM infection and other bacterial infections on mortality in ICU patients with COVID-19 during the pandemic. In addition, the light sensitivity of the phenotypes occurring in this period and the frequency and types of infection were assessed.

## 2 Methods

### 2.1 Study design and settings

The single-center cross-selection study was conducted from September 2020 to September 2022 at the Intensive Care Unit 4 Military Hospital of Wroclaw, Poland. The study was registered in the Australian New Zealand Clinical Trials Registry (ANZCTR) with registration no. ACTRN12621001300864. The stands for The Strengthening the Reporting of Observational Studies in Epidemiology (STROBE) were followed and the STROBE checklist was used for enrolment and allocation of patients (von Elm et al., 2007).

### 2.2 Study participants

**Figure 1:**
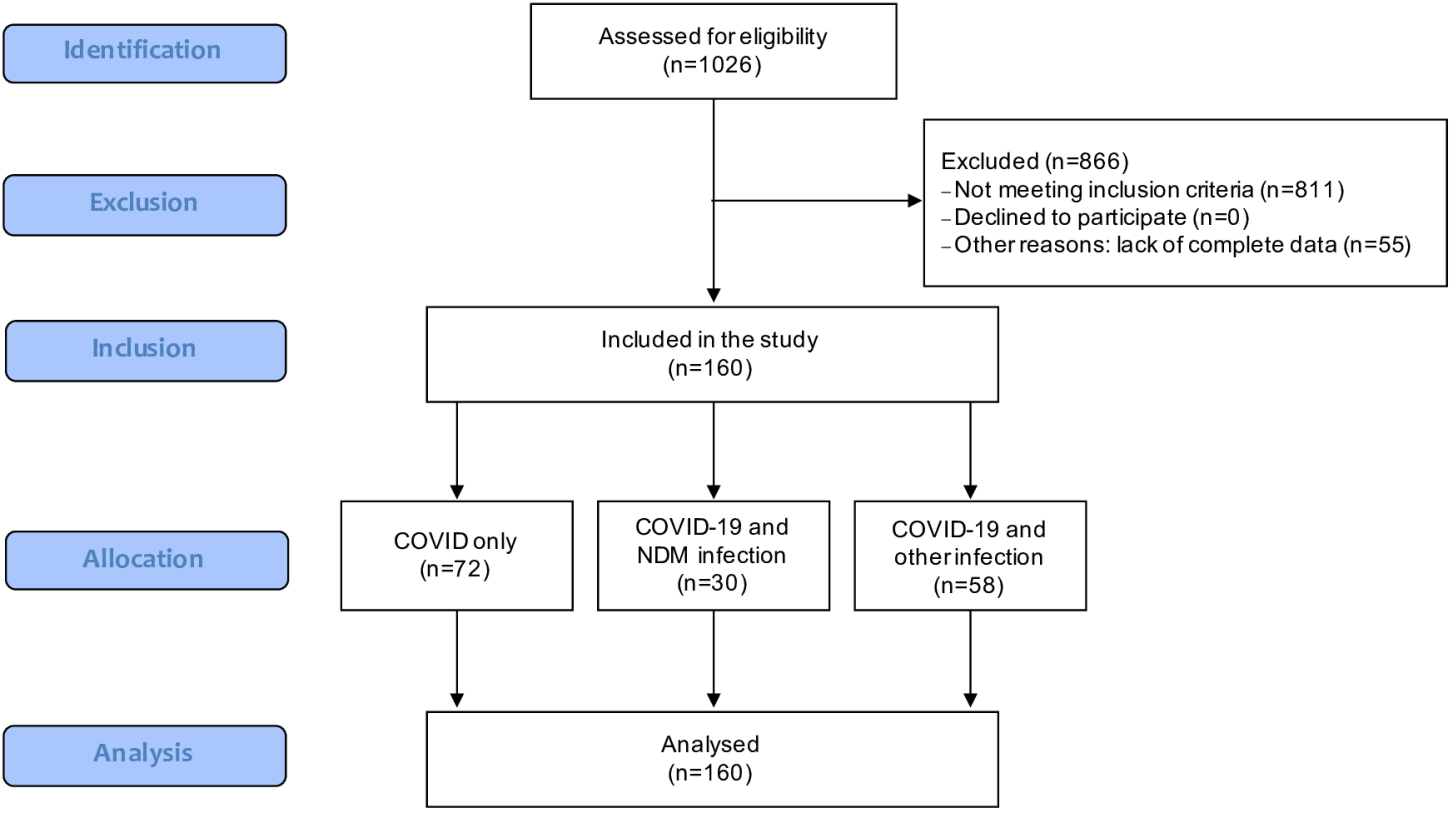
Flow-chart of study participants (STROBE).

The stratification of patients participating in the study is shown in the Flow-chart (**Figure 1**).

Adult patients with clinical symptoms and lab-confirmed COVID-19 infections (irrespective of gender) who were treated in the ICU of the 4th Military Clinical Hospital in Wroclaw during the period from September 2020 to September 2022 were enrolled in the present study. All of them were treated in an ICU for COVID-19 pneumonia (N = 160). Three groups were distinguished among these patients: patients with COVID-19 infection only (N = 72; COVID-19 only group), patients with COVID-19 infection and an infection caused by Klebsiella pneumoniae NDM (N = 30; COVID-19 with NDM infection group), and patients with COVID-19 infection and infection with bacterial etiology other than *Klebsiella pneumoniae* NDM (N = 58; COVID-19 with other infection group). RT-PCR tests for COVID-19 were used to diagnose SARS-CoV-2 infections. Patients with test-confirmed COVID-19 infections but without clinical symptoms, those with negative SARS-CoV-2 tests, and those aged below 18 years were excluded from the study.

### 2.3 Intervention

All patients admitted to the intensive care unit underwent a comprehensive set of screening tests (nasal swab, rectal swab) and diagnostic tests appropriate to the site of the ongoing infection (blood, urine, BAL, tissue, wound swab). According to the procedure regarding indications for collecting material for microbiological examinations, samples from the lower respiratory tract should be taken in the case of suspected ventilator-associated pneumonia with concomitant deviations in clinical and radiological findings. Urine cultures were performed in cases of fever with concomitant abnormalities in general urine examination and inflammatory parameter elevation. Immediate blood culture collection was indicated in cases of suspected infection with at least two of the following symptoms and/or parameters: body temperature 38.4–39.4°C, age > 65 years, chills, vomiting, blood pressure drops, leukocyte count >18,000/μl, granulocyte percentage > 80%, platelet count < 15,000/μl, and creatinine > 2 mg/dl. Tissue biopsies or wound swabs were taken only when signs of inflammation were present. The diagnosis of systemic infection was always established based on obtaining a positive microbiological result with concurrent clinical symptoms. Tests were repeated during hospitalization in the event of worsening clinical condition or lack of therapeutic success despite the use of targeted antibiotic therapy.

Infections were diagnosed when positive microbiological results were obtained in the presence of concurrent clinical symptoms. Colonization was identified when MDRO microorganisms were cultured from samples collected as part of screening tests, including nasal and rectal swabs.

### 2.4 Outcomes

The present study aimed to assess the impact of *Klebsiella pneumoniae* NDM infection on mortality in patients with SARS-CoV-2 infection during the pandemic.

### 2.5 Measures

Demographic data (age, weight, and height) were included in the study data. The procalcitonin (PCT), C-reactive protein (CRP), and white blood cell (WBC) levels were determined in the blood samples collected via venous cannula upon hospital admission and on the third, fifth, and seventh days of hospital stay. In addition, the Acute Physiology and Chronic Health Evaluation (APACHE II) and Simplified Acute Physiology Score (SAPS II) scales were used upon admission, and the Sequential Organ Failure Assessment (SOFA) score was assessed each day. The impact of comorbidities such as hypertension (AH), ischemic heart disease (IHD) and diabetes (DM) was taken into account in the analysis (**Table 1**).

**Table 1:** Demographic characteristics of study population.

Variable	Total	COVID-19 and NDM infection	COVID-19 and other infection	COVID-19 only	p-value
Population (*N*)	160	30	58	72	
Gender					
Women, *n* (%)	56 (35%)	8 (27%)	17 (29%)	31 (43%)	0.15**
Men, *n* (%)	104 (65%)	22 (73%)	41 (71%)	42 (57%)	
Age (years)					
*M* ± *SD*	63.6 ± 12.8	65.6 ± 8.7	62.1 ± 15.2	63.9 ± 12.0	0.74*
*Me* [*Q*1; *Q*3]	67 [57; 72]	67 [61; 73]	66 [51; 73]	67 [55; 71]	
*Min* - *Max*	27 – 86	42 - 78	27 – 86	34 – 86	
Body high (cm)					
*M* ± *SD*	171 ± 10	172 ± 9	172.2 ± 9.0	170.4 ± 10.2	0.55*
*Me* [*Q*1; *Q*3]	174 [164; 180]	175 [164; 180]	175 [165; 180]	170 [160; 179]	
*Min* - *Max*	150 - 194	156 - 187	152 – 190	150 – 194	
Body weight (kg)					
*M* ± *SD*	88 ± 22	91 ± 22	88.8 ± 19.7	86.4 ± 24.1	0.37*
*Me* [*Q*1; *Q*3]	85 [73; 100]	90 [76; 101]	88 [74; 100]	83 [70; 97]	
*Min* - *Max*	42 – 193	42 - 170	57 – 148	43 – 193	
BMI (kg/m^2^)					
*M* ± *SD*	30.0 ± 7.0	30.7 ± 6.6	29.9 ± 5.8	29.7 ± 7.9	0.38*
*Me* [*Q*1; *Q*3]	29 [25; 34]	29 [27; 35]	29.2 [25; 35]	28 [24; 33]	
*Min* - *Max*	16 - 65	16 - 50	19 – 43	19 – 65	
AH					
0, *n* (%)	67 (42%)	11 (37%)	25 (43%)	31 (43%)	0.81**
1, *n* (%)	93 (58%)	19 (63%)	33 (57%)	41 (57%)	
IHD					
0, *n* (%)	108 (68%)	20 (67%)	38 (66%)	50 (69%)	0.89**
1, *n* (%)	52 (52%)	10 (33%)	20 (34%)	22 (31%)	
DM					
0, *n* (%)	97 (61%)	19 (63%)	35 (60%)	43 (60%)	0.94**
1, *n* (%)	63 (39%)	11 (37%)	23 (40%)	29 (40%)	

n, number of participants; M, mean; Me, median; Min, minimum value; Max, maximum value; Q1, lower quartile; Q3, upper quartile; p, level of statistical significance.

*Kruskal-Wallis test; **Chi-square test.

AH, Arterial Hypertension; IHD, Ischemic Heart Disease; DM, Diabetes mellitus.

### 2.6 Microbiology procedure

#### 2.6.1 Blood culture

The materials for the study included the blood taken after prior disinfection of the skin based on the principles of collection of material for microbiological tests. The BacT/ALERT automatic blood culture monitoring system (bioMérieux, France) was used in the laboratory. Blood (8–10 ml volume) was collected in bottles intended for the cultivation of microorganisms, as follows: BacT/ALERT FA Plus medium (aerobic medium with antibiotic neutralizer; bioMérieux, France) and BacT/ALERT FN Plus medium (anaerobic medium with antibiotic neutralizer; bioMérieux, France). It was collected using a blood culture kit consisting of a sterile “butterfly” needle connected to a holder. Routinely, blood was drawn into a minimum of two blood culture kits (kit = 1 aerobic bottle + 1 anaerobic bottle). The incubation time was 5 days. When information about a positive sample was obtained from the automatic system, inoculation was done on the following solid media: Columbia agar with 5% sheep blood (bioMérieux, France), chocolate agar (bioMérieux, France), and MacConkey agar (bioMérieux, France). Then the plates were incubated at 35–37°C under aerobic conditions and with the addition of CO_2_.

#### 2.6.2 Urine culture

Urine samples were obtained either through self-administered clean catch or catheterization. The urine was mixed, and a sterile plastic loop (10 μL) was used to inoculate CHROMID^®^ CPS^®^ Elite/Columbia CNA + 5% sheep blood culture media (bioMérieux, France). All the plates were incubated at 37°C for ≥ 18 h and then examined for evidence of growth. Plates with < 10^3^ colony-forming unit (CFU)/μL were reported as normal urogenital flora. Plates with growth (≥ 10^3^ CFU/μL) were subcultured based on the standard procedures for bacterial identification and susceptibility.

#### 2.6.3 Quantitative culture of bronchial secretion

Another material for the study was a secretion from the lower respiratory tract using bronchoalveolar lavage (BAL). Quantitative inoculation of BAL was done on the following culture media: Columbia agar with 5% sheep blood (bioMérieux, France), chocolate agar (bioMérieux, France), MacConkey agar (bioMérieux, France), Mannitol salt agar (bioMérieux, France), and Sabouraud medium with chloramphenicol (bioMérieux, France). The plates were incubated at 35–37°C under aerobic conditions and with the addition of CO_2_.

#### 2.6.4 Wound swabs/biopsy tissue samples/peritoneal fluid

Wound swabs were taken and placed in Amies transport media (bioMérieux, France), and biopsy tissue samples and peritoneal fluid were stored in thioglycollate broth (bioMérieux, France). A semi-quantitative technique was used to assess bacterial growth from the swabs. The samples were inoculated on Columbia agar (bioMérieux, France), chocolate agar (bioMérieux, France), MacConkey agar (bioMérieux, France), Mannitol salt agar (bioMérieux, France), and Sabouraud agar (bioMérieux, France) for overnight incubation at 37°C + 5% CO_2_.

#### 2.6.5 Central catheter tip

Semi-quantitative inoculation of the catheter according to Maki was performed using the following culture media: Columbia agar with 5% sheep blood (bioMérieux, France), MacConkey agar (bioMérieux, France), Mannitol salt agar (bioMérieux, France), and Sabouraud medium with chloramphenicol (bioMérieux, France). The plates were incubated at 35–37°C under aerobic conditions.

#### 2.6.6 Rectal swabs

Rectal swabs for carbapenemase-producing bacilli were taken and placed in Amies transport media (bioMérieux, France); inoculated on CHROMID® CARBA SMART agar (bioMérieux, France), CHROMID® ESBL agar (bioMérieux, France), CHROMID® VRE agar (bioMérieux, France), and CHROMID® MRSA SMART (bioMérieux, France); and incubated aerobically at 35–37°C for 24 h.

#### 2.6.7 Nasal swabs

Nasal swabs for MRSA screening were taken and placed in Amies transport media (bioMérieux, France), inoculated on CHROMID^®^ MRSA SMART (bioMérieux, France), and incubated aerobically at 35–37°C for 24 h.

#### 2.6.8 Identification and drug susceptibility

When a homogeneous bacterial culture had been obtained, the microorganisms were identified, and their drug susceptibility was determined, using the VITEK automated system (bioMérieux, France). If necessary, drug susceptibility could be tested manually using concentration gradient strips (Liofilchem, Italy) and a MuellerHinton II medium (bioMérieux, France). The sensitivity level of the cultured strains to colistin was determined using the commercial ComASP Colistin test (Liofilchem, Italy). The results of the susceptibility tests were interpreted according to the current criteria of the European Committee on Antimicrobial Susceptibility Testing (EUCAST) (2021, 2022).

#### 2.6.9 Assessment of carbapenemase production

For all bacterial strains resistant to carbapenems, the type of carbapenemase produced was determined using the commercial test RESIST-5 O.O.K.N.V. (CORIS BioConcept, Belgium), and phenotypic methods were used. In addition, strains from infections were sent to the National Reference Center for Microbial Antimicrobial Susceptibility (KORLD) for confirmation.

### 2.7 Ethical considerations

The study protocol was approved by the Bioethics Committee at The Military Medical Chamber in Warsaw, Jelinka 48 str., 01-646 Warsaw, Poland (approval no.: KB–3/21, approval date: 21.05.2021). The study was carried out in accordance with the guidelines of the Declaration of Helsinki and Good Clinical Practice. Informed and written consent was obtained from all the patients.

### 2.8 Sample size

Based on the preliminary analysis (COVID-19 only group n=72 vs. COVID-19 and NDM infection group n=30) of the number of events comparisons, the sample size was calculated. The following parameters were used to calculate the sample size: HR = 0.6, alpha = 0.05; power =0.8. In addition, the Bonferroni correction was included. Based on these parameters, the sample size was set at 160 patients (including a 10% dropouts and refusals). The required number of events for the log-rank test are calculated according to Schoenfeld’s formula (Schoenfeld, 1983).

### 2.9 Statistical analysis

The calculations were carried out using STATISTICA v. 13.3 software (TIBCO Software Inc., USA). The qualitative variables were expressed as frequencies (n) and percentages (%). The Fisher’s exact test was utilized to assess the association between qualitative variables. The quantitative variables (SOFA, APACHE II, SAPS II, PCT, CRP, WBC, LYMPH, NEUT, NLR) were presented as median values (Me) and quartiles (Q1, Q3) in the tables. The Kruskal-Wallis test was employed to determine the significance of differences in means between the COVID-19 and NDM infection, COVID-19 and other infection, and COVID-19 only groups. The overall survival (OS) was evaluated using the Kaplan-Meier method, and the log-rank test was used to compare the survival curves. The multivariate analysis was performed using the Cox proportional hazard regression model. A p-value less than 0.05 was considered to be statistically significant.

## 3 Results

### 3.1 Study group characteristics

The demographic data of the study population, divided into the COVID-19 with NDM infection, COVID-19 with other infection, and COVID-19 only groups, are shown in **Table 1**. The p-values represent the significance levels of the differences between the groups. There were no significant differences in age, body height, body weight, or body mass index between the groups.

No statistically significant differences were found in the groups’ scores (APACHE II, SAPS II, SOFA—1, 3, 5, and 7 days), morphological test results (WBC, LYM, NEUT, TC, NRL), or biochemical test results (CRP, PCT) repeated at 1, 3, 5, and 7 days (**Table 2**).

**Table 2:** Comparison of selected morphological, biochemical, and score tests between the group of: COVID-19 and NDM infection, COVID-19 and other infection and COVID-19 only.

Variables	Day	Total N = 160	COVID-19 and NDM infection	COVID-19 and other infection	COVID-19 only	p-value
		Me (Q1; Q3)	Me (Q1; Q3)	Me (Q1; Q3)	Me (Q1; Q3)	
APACHE II	1	19.0 (15.0; 25.0)	21.5 (19.0; 27.0)	18.0 (15.0; 22.0)	19.0 (15.0; 27.5)	0.11
SAPS II	1	50.5 (37.5; 66.0)	49.5 (40.0; 70.0)	49.0 (36.0; 59.0)	51.5 (37.0; 68.0)	0.41
SOFA	1	8.0 (5.0; 11.0)	9.5 (7.0; 11.0)	7.5 (5.0; 11.0)	8.0 (5.0; 11.0)	0.19
	3	10.0 (7.0; 12.0)	11.0 (9.0; 12.0)	9.0 (7.0; 12.0)	10.0 (7.0; 13.0)	0.39
	5	10.0 (6.0; 13.0)	11.0 (8.0; 13.0)	10.0 (7.0; 12.0)	11.0 (5.0; 14.0)	0.47
	7	10.0 (7.0; 14.0)	12.0 (8.0; 14.0)	10.0 (7.0; 13.0)	10.0 (4.0; 13.0)	0.33
WBC (x10^3/µl)	1	14.0 (9.4; 19.4)	14.2 (10.2; 19.3)	14.0 (9.3; 19.8)	13.4 (8.9; 19.6)	0.98
	3	12.9 (9.4; 18.1)	11.7 (9.5; 15.9)	13.0 (9.0; 20.9)	13.5 (10.6; 19.2)	0.61
	5	13.9 (9.5; 19.8)	13.1 (9.0; 20.1)	13.7 (9.5; 17.8)	14.8 (10.1; 19.9)	0.79
	7	13.3 (10.5; 18.1)	12.7 (10.7; 17.0)	14.4 (10.3; 18.6)	12.6 (10.5; 16.2)	0.63
LYM (%)	1	5.6 (3.3; 8.2)	5.5 (4.3; 7.9)	4.7 (2.7; 7.5)	6.0 (3.7; 8.8)	0.18
	3	5.9 (3.4; 8.5)	5.2 (3.4; 8.0)	5.0 (2.9; 8.0)	6.3 (3.9; 8.8)	0.60
	5	5.8 (4.1; 9.2)	5.8 (4.5; 8.3)	5.5 (2.7; 9.2)	6.4 (4.2; 10.4)	0.38
	7	6.2 (3.5; 10.1)	5.5 (4.0; 8.0)	5.4 (2.8; 9.2)	8.0 (4.8; 12.1)	0.11
NEUT (%)	1	88.0 (82.7; 91.2)	87.9 (85.0; 91.8)	88.8 (83.0; 91.0)	87.3 (82.2; 91.2)	0.44
	3	86.8 (82.0; 90.8)	89.1 (84.5; 92.0)	87.7 (80.0; 90.6)	86.2 (82.0; 90.0)	0.17
	5	85.0 (78.2; 89.6)	85.8 (80.8; 90.2)	85.5 (78.4; 90.7)	83.9 (77.0; 87.3)	0.22
	7	83.8 (77.7; 88.8)	85.8 (79.3; 89.8)	84.1 (78.1; 90.4)	81.0 (77.0; 85.0)	0.08
NLR	1	15.6 (10.4; 26.9)	16.2 (10.4; 21.5)	19.0 (11.2; 34.6)	14.0 (9.8; 24.4)	0.18
	3	14.9 (9.9; 27.3)	17.3 (11.0; 27.0)	17.3 (10.4; 31.9)	13.7 (9.6; 23.7)	0.56
	5	14.8 (9.1; 22.0)	15.3 (10.0; 19.2)	15.6 (9.3; 35.0)	12.8 (7.4; 20.4)	0.34
	7	13.9 (8.3; 24.2)	16.0 (10.3; 23.0)	15.9 (8.7; 33.3)	9.7 (6.3; 17.8)	0.10
PLT (x10^3/µl)	1	235.5 (160.5; 328.0)	216.5 (165.0; 291.0)	246.0 (165.0; 341.0)	237.5 (143.0; 317.0)	0.55
	3	241.0 (148.0; 317.0)	265.0 (177.5; 320.0)	234.0 (168.0; 323.0)	240.0 (141.0; 302.0)	0.50
	5	243.5 (159.0; 329.0)	270.5 (163.0; 348.5)	226.5 (149.0; 277.0)	250.0 (156.5; 334.0)	0.46
	7	229.5 (162.5; 313.5)	293.0 (186.0; 334.0)	206.0 (142.0; 296.0)	266.5 (176.0; 354.0)	0.05
CRP (mg/L)	1	154.5 (68.7; 230.0)	160.0 (104.0; 231.0)	124.8 (61.9; 220.0)	164.0 (66.9; 230.0)	0.46
	3	120.0 (71.7; 196.0)	139.1 (84.5; 205.0)	111.4 (55.0; 191.0)	126.5 (88.5; 209.0)	0.22
	5	137.8 (60.7; 224.0)	165.0 (102.5; 273.5)	123.4 (67.0; 213.0)	146.9 (44.9; 216.9)	0.18
	7	150.0 (70.0; 225.0)	172.5 (81.0; 297.0)	137.0 (69.0; 224.0)	140.5 (70.0; 222.0)	0.60
PCT (ng/mL)	1	0.7 (0.2; 2.1)	1.0 (0.3; 3.4)	0.7 (0.2; 1.4)	0.7 (0.2; 2.3)	0.21
	3	0.6 (0.2; 2.5)	0.8 (0.2; 3.5)	0.5 (0.1; 2.3)	0.7 (0.2; 2.3)	0.47
	5	0.5 (0.2; 1.7)	0.9 (0.2; 2.8)	0.5 (0.1; 1.5)	0.5 (0.2; 1.2)	0.35
	7	0.8 (0.2; 2.2)	1.4 (0.4; 3.9)	0.6 (0.2; 1.7)	0.5 (0.1; 1.8)	0.16

SOFA, the Sequential Organ Failure Assessment score; APACHE II, the Acute Physiology and Chronic Health Evaluation II score; SAPS II; WBC, White Blood Cells; LYM, Lymphocytes; NEUT, Neutrophils; NLR, neutrophil to lymphocyte ratio; PLT, platelets; CRP, C-reactive protein; PCT, procalcitonin.

No statistically significant differences were found in the groups regarding comorbidities (AH, IHD, DM) (**Table 1**).

Median length of hospitalization in the overall group (N = 160) and in the COVID-19 with other infection and COVID-19 only groups was 9 days; median length of hospitalization in the COVID-19 with NDM infection group was 12.5 days. (**Table 3**).

**Table 3:** Length of hospitalization.

Length of stay (days)	Me	Q1	Q3
Total n=160	9	5	14
COVID-19 and NDM infection	12,5	7	19,75
COVID-19 and other infection + COVID-19 only	9	5	13

n, number of participants; Me, median; Q1, lower quartile; Q3, upper quartile.

### 3.2 Mortality

**Figure 2:**
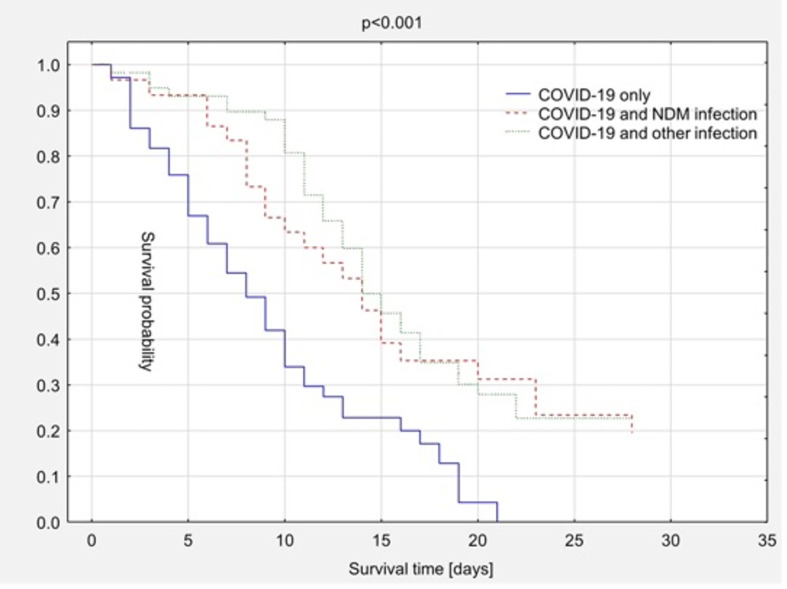
Kaplan-Meier survival curves – comparison depending on additional bacterial infection.

The overall survival rates of the COVID-19 with NDM infection, COVID-19 with other infection, and COVID-19 only groups are compared in **Figure 2**. Bacterial infection, including with *Klebsiella pneumoniae* NDM type, did not elevate mortality rates. In the group of patients who survive the acute phase of COVID-19 the prolonged survival time was demonstrated. The median overall survival time was 13 days in the COVID-19 with NDM infection group, 14 days in the COVID-19 with other infection group, and 7 days in the COVID-19 only group (**Figure 2**).

### 3.3 Independent predictors of outcomes

Both univariate and multivariate Cox proportional hazard regression models were used in the present study to examine the factors linked to mortality. The factors found to be significant in both the univariate and multivariate analyses were regarded as being associated with mortality (as indicated in **Table 4**). The results of the multivariate analysis revealed that age (HR = 1.017; p = 0.046), APACHE II score (HR = 1.042; p ≤ 0.001), and CRP (HR = 1.002; p = 0.008) were predictors of mortality in all the groups. Comparing the COVID-19 with NDM infection and COVID-19 only groups, the adjusted model estimated a statistically significant hazard ratio of 0.28 (p = 0.002), indicating that the patients in the COVID-19 with NDM infection group had a lower 28-day mortality rate (**Table 4**).

**Table 4:** Univariate and multivariate Cox regression analysis of risk factors influence on 28-days mortality.

Variables		Univariate model			
		HR	95% CI		p-value
Age		1.024	1.009	1.039	0.002
APACHE II		1.039	1.019	1.059	0.000
SAPS II		1.022	1.013	1.031	0.000
SOFA		1.111	1.060	1.164	0.000
WBC (x10^3/µl)		1.024	1.001	1.046	0.039
LYM (%)		0.999	0.968	1.030	0.937
NEUT (%)		1.002	0.978	1.026	0.885
NLR		0.998	0.993	1.004	0.514
PLT (x10^3/µl)		0.998	0.996	1.000	0.036
CRP (mg/L)		1.002	1.001	1.004	0.006
PCT (ng/mL)		1.008	1.003	1.013	0.001
Sex (ref. M)		1.102	0.750	1.619	0.621
NDM (ref. non-NDM)		0.751	0.473	1.191	0.223
Bacterial infection group (ref. COVID-19 only)	COVID-19 and NDM infection	0.421	0.252	0.702	0.122
	COVID-19 and other infection	0.370	0.241	0.569	0.006
		Multivariate model 1			
Age		1.018	1.002	1.034	0.032
APACHE II		1.037	1.016	1.059	0.001
CRP (mg/L)		1.002	1.001	1.004	0.005
NDM (ref. non-NDM)		0.526	0.324	0.853	0.009
		Multivariate model 2			
Age		1.017	1.000	1.034	0.046
APACHE II		1.042	1.020	1.063	0.000
CRP (mg/L)		1.002	1.001	1.004	0.008
Bacterial infection group (ref. COVID-19 only)	COVID-19 and NDM infection	0.280	0.161	0.486	0.002
	COVID-19 and other infection	0.365	0.234	0.569	0.075

HR, hazard ratio; CI, confidence interval; SOFA, the Sequential Organ Failure Assessment score; APACHE II, the Acute Physiology and Chronic Health Evaluation II score; SAPS II; WBC, White Blood Cells; LYM, Lymphocytes; NEUT, Neutrophils; PLT, platelets; CRP, C-reactive protein; PCT, procalcitonin.

### 3.4 Susceptibility testing of NDM-producing *Klebsiella pneumoniae*

**Figure 3:**
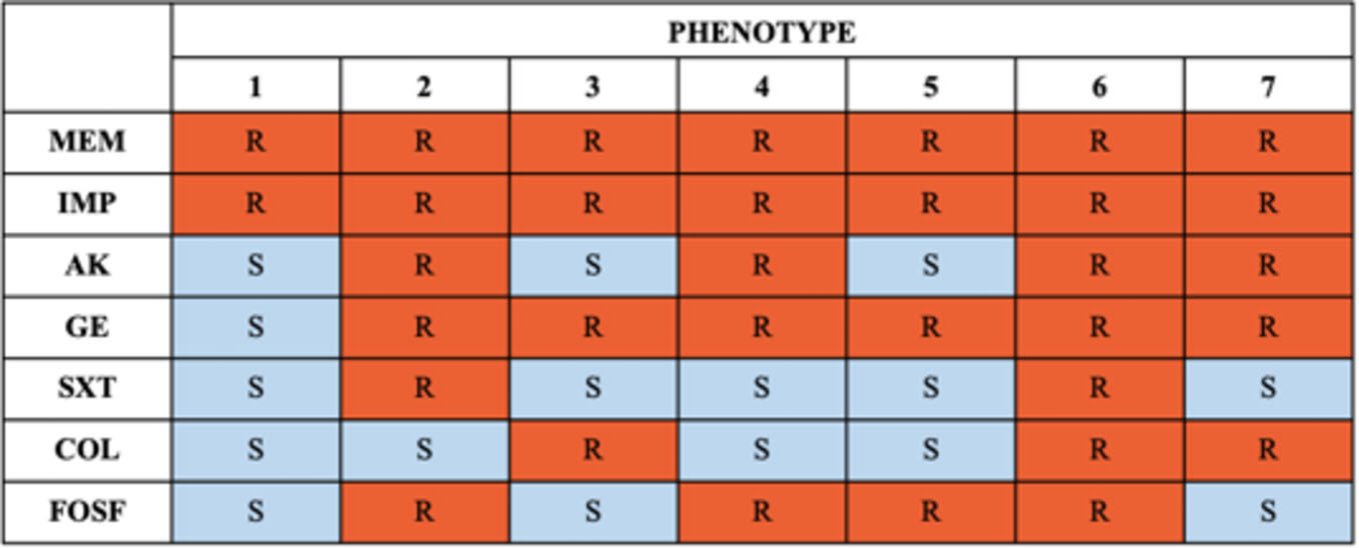
Phenotypes of resistance. IMP, imipenem; MEM, meropenem; AK, amikacin; GE, gentamycin; SXT, trimethoprim/sulfamethoxazole; COL, colistin; FOSF, fosfomycin.

Susceptibility was tested against antibiotics routinely used in the treatment of infections. New therapeutic options, like cefiderocol, were not available in Polish hospitals during the study period. All the identified strains showed resistance to carbapenems. Seventy-five percent of the strains were sensitive to colistin, 84% were sensitive to amikacin, 94% were sensitive to trimethoprim/sulfamethoxazole, and 72% were sensitive to fosfomycin. Only 3% of the tested strains were sensitive to gentamycin. All examined isolates exhibited varied drug resistance. The phenotypic resistance patterns of the analyzed strains are presented in **Figure 3**.

## 4 Discussion

A 2022 report by the European Center for Disease Prevention and Control (2022) highlighted an escalating antimicrobial resistance, especially carbapenem-resistant strain of *Klebsiella pneumoniae*, from 2016 to 2020. The data revealed a concerning trend, and the situation in Poland, as confirmed by the National Reference Center for Antimicrobial Susceptibility Testing of Microorganisms, reflected this issue. The number of confirmed cases of carbapenemase-producing *Enterobacterales* strains surged from 2,064 in 2019 to 4,172 in 2021. Specifically, cases of NDM strains from infections increased from 1,527 in 2019 to 3,036 in 2021 (Hryniewicz et al., 2022).

Preventing the spread of multidrug-resistant strains, especially in ICUs, is a great challenge. In addition, during the COVID-19 pandemic, isolating patients with COVID-19 and ensuring an appropriate sanitary regime were significant challenges due to the need to use PPE. There was a rapid spread of infections caused by Gram-negative rods of the Enterobacterales family in this group of patients. Under natural conditions, these microorganisms colonize the digestive tract, being a component of the physiological flora (Pratt et al., 2001; Pelfrene et al., 2021).

In immunocompromised patients with accompanying lymphopenia (e.g., caused by SARS-CoV-2 infection), Gram-negative bacteria can cause respiratory infections, urinary tract infections, soft-tissue infections, and postoperative wound infections, including sepsis and septic shock. Carbapenem-resistant bacilli are currently the biggest clinical problem. The mechanisms responsible for the development of resistance are a decrease in the permeability of the outer sheaths with the simultaneous increased production of extended-spectrum beta-lactamases (ESBLs) or AmpC cephalosporins, as well as carbapenemase-type β-lactamases (Nordmann et al., 2011, 2012; European Centre for Disease Prevention and Control (ECDC), 2017).

During the COVID-19 pandemic, from September 2020 to the end of September 2022, the 4th Military Clinical Hospital in Wroclaw served as a center for the treatment of patients with COVID-19. It ensured the availability of 160 internist beds with an infectious profile, including 16 ICU beds. It provided security for patients with SARS-CoV-2 infection requiring surgery in the fields of neurosurgery, urology, general surgery, laryngology, maxillofacial surgery, vascular surgery, oncology surgery, orthopedics, traumatology, and cardiac surgery. Overall, 1026 patients were hospitalized in the ICU in the analyzed period of which 160 were being treated for COVID-19. Despite the isolation of COVID-19 patients with confirmed infection and the elimination of additional risk factors for infection (e.g., restrictions on the movement of personnel, modification of airlocks, special supervision of hand decontamination, and double protection by protective clothing), an increase in the number of super-infections was observed. In the group of 160 ICU patients treated for COVID-19, *Klebsiella pneumoniae* ESBL or NDM strains were observed. The *Klebsiella* species are among the top 10 pathogens causing nosocomial bacterial infections (Mahmud et al., 2022). No *Klebsiella pneumoniae* infection carbapenemase- (KPC-), VIM-, or IMP-type cases was reported in the aforementioned period.

In our study the percentage of infections caused by CRE in patients on ICU with COVID-19 during the pandemic was 18.75%. According to available publications, the prevalence of CPE in patients with COVID-19 ranges between 0.35% and 54 (Lai et al., 2020; Mędrzycka-Dąbrowska et al., 2021; Yahya, 2022). According to Tiri et al. (2020), the number of cases during the COVID-19 pandemic increased from 6.7% to 50% of the patients treated in the ICU.

In the present study, three groups of ICU patients were extracted: those with NDM bacterial infection, those with other bacterial infection, and those with “pure” COVID-19. The estimated 28-day mortality rate in the whole group of ICU patients was 71.25%. This high mortality rate resulted from the severe course of COVID-19 in most patients in the first days after the onset of the disease. In particular, elderly patients with COVID-19 (COVID-19 only group) who were admitted in a serious condition to the ICU from intermittent wards, where steroids, antibiotics, non-invasive ventilation, prone position, and invasive ventilation had previously been used, which significantly limited their chances of survival. The patients who survived the first phase of the disease developed a bacterial infection. In the COVID-19 only group, there were 72 patients with a median survival time of 7 days. The patients in the COVID-19 and NDM infection and COVID-19 and other infection groups had an average survival time of 13 or 14 days. Fast identification of the pathogen and application of targeted treatment on ICU were shown to be major factors limiting mortality in the case of bacterial infection in the course of COVID-19.

The prolonged survival observed in the bacterial infection groups is hypothesized to be influenced by extended hospitalization durations, thereby establishing an association with a heightened risk of developing multidrug-resistant infections. This suggests a potential relationship between prolonged hospital stays, increased susceptibility to infections, and the subsequent development of multidrug-resistant infections. Patients who survived the acute phase of COVID-19 demonstrated an increased susceptibility to subsequent infections, potentially attributed to prolonged hospitalizations and, consequently, extended survival periods. This observation suggests a correlation between prolonged hospital stays, increased vulnerability to infections, and extended overall survival in this patient cohort.

The results of the multivariate analysis in the present study revealed that age (HR = 1.017; p = 0.046), APACHE II score (HR = 1.042; p ≤ 0.001), and CRP (HR = 1.002; p = 0.008) were predictors of mortality in all the patient groups.

In previous research, COVID-19-associated bacterial infections have resulted in increased mortality, particularly in the group with *Klebsiella pneumoniae*-type NDM infection (Said et al., 2022). In our study, no increase in mortality was demonstrated in the group of patients with additional infections (COVID-19 with NDM infection and COVID-19 with other infection groups). This finding differs from the findings presented in existing publications.

Based on the available data, the variant of the virus causing the infection also influenced mortality in the course of COVID-19. The lowest survival rate was observed during infection with the Delta variant (Lavrentieva et al., 2023). The spread of the Delta variant, which was more transmissible than the previously dominant Alfa variant, in the autumn of 2021 caused the fourth wave of the COVID-19 pandemic in Europe, including Poland. In the early autumn of 2021, the Delta variant of the SARS-CoV-2 virus dominated in Poland. According to the GISDAD EpiCoV database data, as of October 20, 2021, it accounted for over 97% of the SARS-CoV-2 sequences reported from Poland within seven days. According to European Center for Disease Prevention and Control data, in the 42nd week of 2021, the Delta variant accounted for 99.9% of SARS-CoV-2 infections in Poland. In addition, in the fourth wave of the pandemic, a high mortality rate of 62.9% was observed.

### 4.1 Study limitations

The present study had several limitations. First, it was a single-center study thus the results may not be representative of broader populations or diverse settings. Second, due to the observational nature of the study, several unmeasured confounders could have affected the outcomes. We focused on bacterial infection and ICU mortality. More data on the specific causes of death in COVID-19 could help interpret mortality rates better. The variant responsible for the infection was also not studied, which could have affected mortality.

## 5 Conclusions

In patients treated for SARS-CoV-2 infection acquiring a bacterial infection due to prolonged hospitalization associated with the treatment of COVID-19 did not elevate mortality rates. The data suggests that in severe COVID-19 patients who survived beyond the first week of hospitalization, bacterial infections, particularly Klebsiella pneumoniae NDM, do not significantly impact mortality. Multivariate analysis revealed that age, APACHE II score, and CRP were predictors of mortality in all the patient groups.

## Data availability statement

The raw data supporting the conclusions of this article will be made available by the authors, without undue reservation.

## Ethics statement

The studies involving humans were approved by Bioethics Committee at The Military Medical Chamber in Warsaw, Jelinka 48 str., 01-646 Warsaw, Poland (approval no.: KB–3/21, approval date: 21.05.2021). The studies were conducted in accordance with the local legislation and institutional requirements. The participants provided their written informed consent to participate in this study.

## Author contributions

JJ: Conceptualization, Data curation, Formal analysis, Funding acquisition, Investigation, Methodology, Software, Supervision, Visualization, Writing – original draft. NS: Conceptualization, Data curation, Formal analysis, Investigation, Methodology, Project administration, Software, Visualization, Writing – original draft. AW: Data curation, Formal analysis, Investigation, Visualization, Writing – review & editing. LŁ: Formal analysis, Software, Writing – review & editing. MC: Formal analysis, Writing – review & editing. PL: Conceptualization, Data curation, Formal analysis, Funding acquisition, Investigation, Methodology, Project administration, Software, Visualization, Writing – original draft.
